# Effects of the STAMP-inhibitor asciminib on T cell activation and metabolic fitness compared to tyrosine kinase inhibition by imatinib, dasatinib, and nilotinib

**DOI:** 10.1007/s00262-022-03361-8

**Published:** 2023-01-05

**Authors:** Lukas Häselbarth, Axel Karow, Kristin Mentz, Martin Böttcher, Oisin Roche-Lancaster, Manuela Krumbholz, Regina Jitschin, Dimitrios Mougiakakos, Markus Metzler

**Affiliations:** 1 Department of Pediatrics and Adolescent Medicine, University Hospital Erlangen, Erlangen, Germany; 2 Comprehensive Cancer Center Erlangen-European Metropolitan Area Nuremberg (CCC-ER-EMN), Nuremberg, Germany; 3 Interdisciplinary Centre for Clinical Research (IZKF), Erlangen, Germany; 4 Department of Internal Medicine 5, Hematology and Oncology, University Hospital Erlangen, Erlangen, Germany; 5 Department of Internal Medicine, Hematology and Oncology, University Hospital Magdeburg, Magdeburg, Germany; 6 Center of Medical Information and Communication Technology, University Hospital Erlangen, Erlangen, Germany

**Keywords:** CML, TKI, Asciminib, T cell, Activity, Metabolism

## Abstract

T cell function is central to immune reconstitution and control of residual chronic myeloid leukemia (CML) cells after treatment initiation and is associated with achieving deep molecular response as a prerequisite for treatment-free remission, the ultimate therapeutic goal in CML. ATP-pocket-binding tyrosine kinase inhibitors (TKIs) like imatinib, dasatinib, and nilotinib are widely used for treating CML, but they have shown to inhibit T cell function as an “off-target” effect. Therefore, we tested asciminib, the first-in-class BCR::ABL1 fusion protein inhibitor specifically targeting the ABL myristoyl pocket (STAMP) and compared its effects on T cell function with imatinib, dasatinib, and nilotinib. Whereas all four TKIs inhibited the expression of the co-stimulatory protein CD28, the amino acid transporter CD98, proliferation, and secretion of pro-inflammatory cytokines IFNγ, IL-6, and IL-17A upon T cell stimulation, asciminib had less impact on PD-1, activation markers, and IL-2 secretion. T cells treated with asciminib and the other TKIs maintained their ability to mobilize their respiratory capacity and glycolytic reserve, which is an important surrogate for metabolic fitness and flexibility. Overall, we found milder inhibitory effects of asciminib on T cell activation, which might be beneficial for the immunological control of residual CML cells.

**Supplementary Information:**

The online version contains supplementary material available at 10.1007/s00262-022-03361-8.

## Introduction

The introduction of imatinib and the following second generation tyrosine kinase inhibitors (TKIs) dasatinib and nilotinib, targeting the pathognomonic BCR::ABL1 fusion protein, has revolutionized chronic myeloid leukemia (CML) treatment. Despite major advantages of TKI-treatment, a significant proportion of patients develop resistance to targeted therapy and experience substantial TKI side effects. Therefore, it is important to achieve rapid and deeper molecular responses (DMRs), the prerequisite for treatment-free remission (TFR). In addition to the effective suppression of the constitutively activated BCR::ABL1 kinase, the immunological control of resistant BCR::ABL1 stem cells is crucial for achieving stable remission. T cells are important for TFR since they control residual CML cells [1] and induced antileukemia responses in CML patients in remission [2]. That immunological control by T cells is important for CML treatment could be shown at patients in relapse after allogeneic stem cell transplantation which were treated by donor lymphocyte infusion [1]. At CML diagnosis, the immune system is dysfunctional as reflected by increased aberrant immune-inhibitory responses and deficient responses of cytotoxic T lymphocytes (CTL) to leukemia-associated antigens [3]. Furthermore, immune restoration under TKI-therapy is characterized, e.g., by an increased proportion of innate marker expressing CD8^+^ T cells associated with the achievement of DMR and TFR [4]. However, T cell function is inhibited as an off-target effect of ATP-pocket binding TKIs due to the expression of ABL1 by T cells and the broad target spectrum of TKIs [5]. These off-target effects include reduced proliferation, activation, and pro-inflammatory cytokine secretion [6–11].

A novel agent developed to overcome kinase domain resistance mutations is asciminib, which is the first-in-class BCR::ABL1 fusion protein inhibitor that specifically targets the ABL myristoyl pocket (STAMP) [12]. Here, we investigated whether asciminib has less impact on T cell function due to its different target sites compared to the previous generations of ATP-pocket binding TKIs. We studied the effects on T cells in terms of central (activation) markers (CD25, CD28, CD69, CD98, CD137, and PD-1), proliferation, and production of pro-inflammatory cytokines (IFNγ, IL-2, IL-6, and IL-17A) in response to triggering of the T cell receptor and co-stimulatory molecules. Additionally, we investigated the effect of asciminib and conventional TKIs on the metabolic fitness, which is central to the function and development of T cells [13].

## Material and methods

### T cell isolation

Peripheral blood mononuclear cells (PBMCs) were isolated from fresh specimens of healthy donors via Ficoll-Paque (GE Healthcare, Chicago, USA) and cryopreserved. T cells were purified from PBMCs via negative selection using the human Pan T cell isolation kit (Miltenyi Biotec, Bergisch Gladbach, Germany) or the human EasySep™ T cell isolation kit (StemCell, Vancouver, Kanada) according to manufacturer’s instructions. Purity of cells was checked with an αCD3-APC-antibody.

### Cell culture

T cells were cultured at a density of 1–1.75 · 10^6^/mL in RPMI1640, supplemented with 10% FCS (ThermoFisher Scientific, Waltham, USA), 2 mM L-Glutamine (Sigma-Aldrich, St. Louis, USA) and 40 U/ mL Penicillin–Streptomycin (P/S, ThermoFisher Scientific) and treated with/without TKIs for 72 h (96 h for measurement of activation markers) at 37 °C and 5% CO_2_. For T cell stimulation activation/expansion beads, coated with αCD2/3/28 (Miltenyi Biotec) at a bead-T cell ratio of 1:2 were added simultaneously to TKIs or for activation marker measurements 72 h after TKI-treatment for 24 h. Three different concentrations for each TKI were chosen in accordance with in vitro data of T cells [7, 8, 14, 15] and with in vivo serum-/plasma concentrations of CML patients [12, 16–20]: Asciminib (MedChemExpress, Monmouth Junction, USA) 1.25, 5, and 10 µM, imatinib (Sigma-Aldrich) 2.5, 5, and 10 µM, dasatinib (Sellekchem, Houston, USA) 2.5, 5, and 10 nM, and nilotinib (StemCell) 1.25, 2.5, and 5 µM.

### Analysis of TKI-toxicity, proliferation, and protein expression

T cells were examined using a FACS Canto II flow cytometer (BD Biosciences, New Jersey, USA) and subsequently analyzed with the FlowJo software Version 10.6.2 (TreeStar, Ashland, USA). Cells were manually gated for 20.000 single living T cells per sample or 10.000 CD8+ T cells per sample if αCD4-/αCD8-stainings were included. For viability measurements also dead cells were included in the analysis. The toxicity of TKIs was measured via Annexin V-FITC (Biolegend, San Diego, USA) and 7-Aminoactinomycin (7AAD, Biolegend). Annexin V-FITC and 7AAD were incubated for 10–15 min at RT. Cells negative for Annexin V and 7AAD were characterized as vital.

Washing steps for proliferation, surface- and activation markers were carried out using FACS-puffer, containing PBS (ThermoFisher Scientific), supplemented with 0.25% bovine serum albumin (Carl Roth, Karlsruhe, Germany). Antibodies were incubated for 20 min at 4 °C. All used antibodies are listed in supplementary table 1. Proliferation was measured using Violet Proliferation Dye 450 (VPD450, BD Biosciences). As a proliferation parameter, the division index was calculated, which describes the average division of all cells, including the undivided cells.

### RNA-expression of T cell genes

RNA of stimulated T cells, treated with medium TKI concentrations (imatinib: 5 µM, dasatinib: 5 nM, nilotinib: 2.5 µM, asciminib: 5 µM), was isolated with the innuPREP DNA/RNA Mini Kit (Analytik Jena GmbH, Jena, Germany) according to the manufacturer’s instructions. RNA library preparation and sequencing were performed by Novogene Co. LTD (Cambridge, UK). Data analysis was performed using the software R (R Core Team, version 4.0.4). Data were aligned using the RsubRead package with the GRCh38 primary assembly. The RsubRead function, FeatureCounts, was used to count mapped reads for genomic features and with the DESeq2 package, differentially expressed genes were detected. All data were corrected for batch effects. TKI-induced changes in gene-expression were considered as significant when the |Log twofold-change| was > 1 and the adjusted p-value (padj) was < 0.05.

### Measurement of cytokine secretion

The concentration of secreted cytokines IFNγ, IL-2, IL-6, and IL-17A was determined in the supernatant of stimulated T cells by the respective ELISA MAX™ Human Deluxe Sets (Biolegend) according to manufacturer’s instructions. Absorbance was measured at 450 nm at a Spectra Max M3 device (Molecular Devices, San José, USA) using the software SoftMax Pro 6.4 (Molecular Devices) and corrected by the reference absorption wavelength at 570 nm. Concentrations were extrapolated from the standard curve using Excel™ 2016 (Microsoft, Redmond, USA).

### Measurement of glucose- and fatty acid (FA) uptake by T cells

Glucose uptake was analyzed by 300 µl of 0.3 mM 6-NBDG (ThermoFisher Scientific) after washing the cells with glucose-free RPMI. Cells were incubated for 15 min at 37 °C, 5% CO_2_, and washed 3–4 times with FACS-puffer before recording at the flow cytometer.

To assess FA uptake T cells were washed with FCS-free RPMI, 10 µl of 2.5 µM Bodipy™ FL C_16_ (ThermoFisher Scientific) were added and cells were incubated for 15 min at 37 °C, 5% CO_2_ before performing two washing steps with ice-cold PBS with 2% FCS (ThermoFisher Scientific).

### Analysis of mitochondrial fitness

T cells were stained with TMRE (tetramethylrhodamine, ethyl ester), MitoTracker™ Green FM**,** and MitoSOX™ Red Mitochondrial Superoxide Indicator (all ThermoFisher Scientific) for analyzing mitochondrial membrane potential (ΔΨm), mass, and superoxide generation, respectively, using FACS.

### Metabolic flux analyses

The activity of the mitochondrial respiratory chain and glycolysis was assessed by metabolic flux analyses with an XF96e Extracellular Flux Analyzer as previously described [21]. In this analysis oxidative phosphorylation (oxPhos) and cytosolic glycolysis are characterized by the oxygen consumption rate (OCR) and extracellular acidification rate (ECAR), respectively, which are measured by fluorophores, detecting oxygen and hydrogen-ions. By subsequent addition of metabolic inhibitors and measurement of changes in OCR and ECAR, different metabolic parameters were calculated.

For the mitochondrial stress test (Agilent Technologies, California, USA), the cartridge ports were loaded with 1 µM of the mitochondrial ATP synthase inhibitor oligomycin (Oligo, Sigma-Aldrich), 1.5 µM of the uncoupling agent carbonyl cyanide-4 (trifluoromethoxy) phenylhydrazone (FCCP, Sigma-Aldrich) and 3 µM of the complex I/III oxPhos inhibitors rotenone (Rot, Sigma-Aldrich) and antimycin A (AA, Sigma-Aldrich). While the basal respiration describes the OCR that attributes to oxPhos, maximal respiration of T cells can be mimicked by adding the uncoupling agent FCCP. The spare respiratory capacity is defined by the difference in OCR between maximal and basal respiration.

For the glycolysis stress test (Agilent Technologies), ports were loaded with 10 mM glucose (Agilent Technologies), 1 µM Oligo and 100 mM of 2-deoxy-d-glucose (2-DG, Biomol GmbH, Hamburg, Germany). Basal glycolysis describes the change of ECAR after glucose-addition, while glycolytic capacity additionally counts in the increase of glycolysis after oligomycin-induced oxPhos-inhibition. The glycolytic reserve is defined as the difference between the glycolytic capacity and basal glycolysis. Data were acquired by the WAVE software version 2.6.1 (Agilent Technologies).

### Statistical analysis

Statistical data analysis was performed using GraphPad Prism Version 9 software (GraphPad Software, San Diego, USA). For statistical analysis mostly Friedman tests were performed. For the metabolic flux analysis, the Kruskal–Wallis test was carried out. All tests compared αCD2/3/28-stimulated TKI-treated T cells with αCD2/3/28-stimulated TKI-untreated control. Friedman- and Kruskal–Wallis tests were followed by a Dunn’s multiple comparison test.

## Results

### Asciminib has less impact on the expression of PD-1 and T cell activation markers compared to other TKIs

**Fig. 1:**
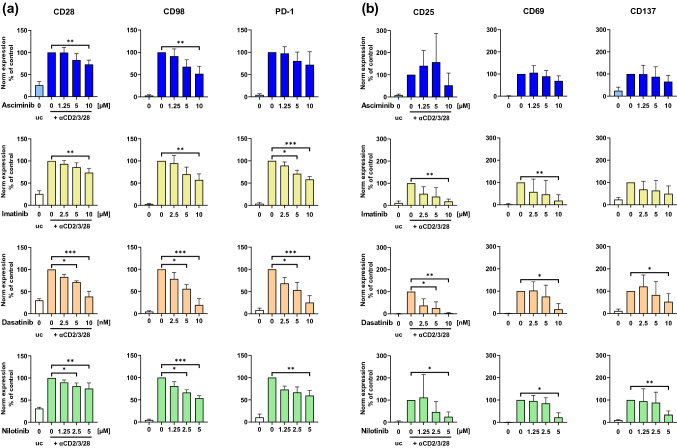
Asciminib has milder inhibiting effects on expression of PD-1 and activation markers CD25 and CD69 than TKIs, targeting the ATP-binding cleft of ABL1. Human T cells were treated with the TKIs asciminib (blue), imatinib (yellow), dasatinib (orange) or nilotinib (green). Expression (MdFI) was measured by flow cytometry (*n* = 5 different donors). **a** Surface marker staining. Treatment with/without TKIs and stimulation with/without αCD2/3/28-beads for 72 h. Subsequent staining with antibodies against CD28, CD98, and PD-1. **b** Activation marker staining. Treatment with/without TKIs for 96 h and in the last 24 h simultaneous stimulation with/without αCD2/3/28-beads. Subsequent staining with antibodies against CD25, CD69, and CD137. Measurements were normalized to the respective TKI-untreated controls. *Norm* normalized, *uc* unstimulated control (no bead addition), ***p < 0.001, **p < 0.01, *p < 0.05

First, we confirmed that the used therapeutic TKI concentrations were subtoxic for CD4+ and CD8+ T cells (Suppl. Figure 1). Asciminib had dose-dependent inhibiting effects on CD28 (73%, SD = 10 (10 µM)) and CD98 (52%, SD = 17 (10 µM)) of αCD2/3/28-stimulated T cells compared to the bead-stimulated control (Fig. 1a). A comparable effect was found upon treatment with imatinib and nilotinib, whereas dasatinib-treated T cells exhibited a lower expression of CD28 (39%, SD = 12) and CD98 (20%, SD = 14) at 10 nM. PD-1-expression under asciminib was not significantly changed (97%, SD = 15 (1.25 µM); 80%, SD = 20 (5 µM); 72%, SD = 29 (10 µM)), while imatinib (58%, SD = 6 (10 µM)), dasatinib (25%, SD = 16 (10 nM)) and nilotinib (60%, SD = 12 (5 µM)) showed significant decreases of expression, especially at the highest concentrations. Similarly, milder effects of asciminib were detected for the early T cell activation marker CD25, with more pronounced donor-specific variations than for the other TKIs (Fig. 1b). Asciminib-treated T cells decreased CD69-expression down to 69% (106%, SD = 32 (1.25 µM); 90%, SD = 26 (5 µM); 69%, SD = 22 (10 µM)), while the expression was reduced below 22% by the other TKIs. Similar to imatinib, CD137 expression was not significantly inhibited by asciminib, while dasatinib (53%, SD = 37 (10 nM)) and nilotinib (34%, SD = 17 (5 µM)) showed inhibitory effects at the highest concentration. Overall, dasatinib exhibited the most significant changes.

On the gene-expression level, only slight TKI-induced effects were observed, indicated by |Log 2 Fold-changes|< 1, with least inhibitory effects of asciminib (Suppl. Figure 2).

### Asciminib affects T cell proliferation and cytokine secretion upon stimulation

**Fig. 2:**
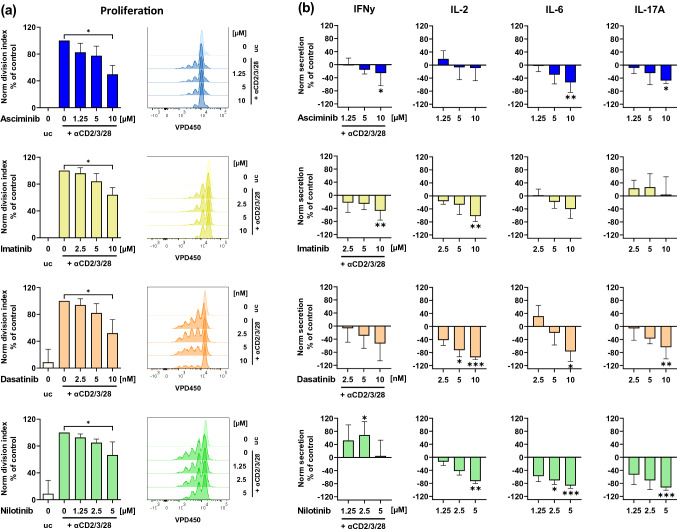
Asciminib interferes with T cell proliferation and secretion of IFNγ, IL-6, and IL17A but not IL-2. Human T cells were treated with the TKIs asciminib (blue), imatinib (yellow), dasatinib (orange) or nilotinib (green) and stimulated with αCD2/3/28-beads for 72 h. **a** Proliferation. VPD450-staining and measurement by FACS (*n* = 5). **b** Cytokine secretion. Supernatants were analyzed for IFNγ, IL-2, IL-6, and IL-17A by ELISA. Measurements were normalized to the respective TKI-untreated controls (= 0% cytokine secretion, *n* = 5–6 different donors). *Norm* normalized, *uc* unstimulated control (no bead addition), ***p < 0.001, **p < 0.01, *p < 0.05

Next, we investigated whether asciminib affects the proliferation of stimulated T cells and its impact on the secretion of critical cytokines. We found that asciminib had inhibiting effects on T cell proliferation, indicated by a halved division index at 10 µM, which was similar to imatinib, dasatinib, and nilotinib (Fig. 2a). Additionally, we found that levels of secreted IFNγ, IL-6, and IL-17A by stimulated T cells were decreased by asciminib at 10 µM compared to the stimulated untreated controls (IFNγ: − 26%, *p* = 0.0417; IL-6: − 53%, *p* = 0.0099; IL-17A: − 47%, *p* = 0.0110, Fig. 2b). Similar results were obtained with imatinib, dasatinib, and nilotinib, except for the IFNγ-levels of nilotinib-treated T cells and IL-17A-levels of imatinib-treated T cells, which largely remained unchanged. Additionally, IL-2-levels did not change by asciminib-treatment, while inhibition of IL-2 secretion by the other TKIs ranged from 62 up to 94% at the maximum concentrations compared to the untreated control.

### Effects of asciminib on glucose/FA uptake and substrate transporter expression of T cells

**Fig. 3:**
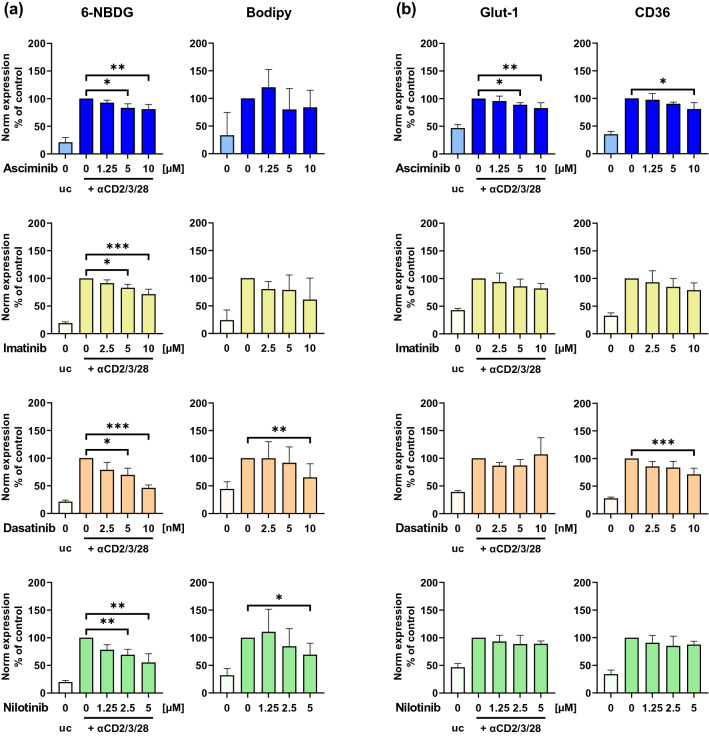
Effects of asciminib on glucose- and FA uptake and Glut-1- and CD36-expression of T cells. Human T cells were treated with/without the indicated concentrations of the TKIs asciminib (blue), imatinib (yellow), dasatinib (orange) or nilotinib (green), stimulated with/without αCD2/3/28-beads for 72 h and analyzed by flow cytometry (*n* = 5 different donors). **a** Glucose- and FA uptake. 6-NBDG staining to measure glucose uptake and Bodipy™ FL C_16_ to measure FA uptake, **b** Glucose- and FA-transporter. αGlut-1 to determine glucose transporter expression and αCD36 to determine LCFA transporter expression. Measurements were normalized to the respective TKI-untreated controls. *Norm* normalized, *uc* unstimulated control (no bead addition), *** *p* < 0.001, **p < 0.01, *p < 0.05

The effects of asciminib on metabolic processes in T cells were examined by the ability of T cells to take up glucose and FA and by assessing the expression of the central glucose transporter Glut-1 and the FA transporter CD36. Glucose uptake was impaired by asciminib, indicated by a decreasing 6-NBDG-signal (Fig. 3a, 93%, SD = 5 (1.25 µM); 83%, SD = 7 (5 µM); 81%, SD = 8 (10 µM)). This expression was higher compared to the other TKIs (imatinib 71%, SD = 9 (10 µM), dasatinib 46%, SD = 5 (10 nM), nilotinib 55%, SD = 16 (5 µM)). Asciminib- and imatinib-induced inhibition of T cell FA uptake was not significant, which was confirmed by Bodipy™ FL C_16_. In contrast, dasatinib and nilotinib showed stronger inhibiting effects with a median fluorescence intensity (MdFI) expression of down to 65%.

Expression of Glut-1 was significantly inhibited by asciminib (89%, SD = 4 (5 µM); 83%, SD = 10 (10 µM)), whereas suppression by the other TKIs was not significant (Fig. 3b). Regarding CD36-expression, asciminib showed a decreasing expression of down to 81% with increasing concentrations, which was higher than the dasatinib-induced expression (71%, SD = 11 (10 nM)), while imatinib- and nilotinib-induced changes were not significant.

### Effects of asciminib on mitochondrial membrane potential, biomass and superoxide generation

OxPhos in the mitochondria is the most important process in the generation of ATP. Therefore, we tested whether important mitochondrial parameters such as the mitochondrial membrane potential ΔΨm, biomass, and superoxide levels of T cells are affected by asciminib, compared to the other TKIs. While a high ∆Ψm is maintained by the energy production during oxPhos, decreases in ∆Ψm are associated with a loss of mitochondrial function. Measuring superoxide levels is an effective way to describe mitochondrial reactive oxygen species (ROS) that are mainly generated by complex I and III of the respiratory chain [22]. Whereas asciminib like imatinib increased ΔΨm at 10 µM (38%, SD = 24 and 56%, SD = 29), dasatinib and nilotinib had no effect (Suppl. Figure 3). The mitochondrial biomass remained stable, independent of TKI-type and –concentration, while there was a non-significant increase of mitochondrial superoxide levels at 10 µM asciminib.

### Asciminib increases the T cells’ respiratory and glycolytic reserve.

**Fig. 4:**
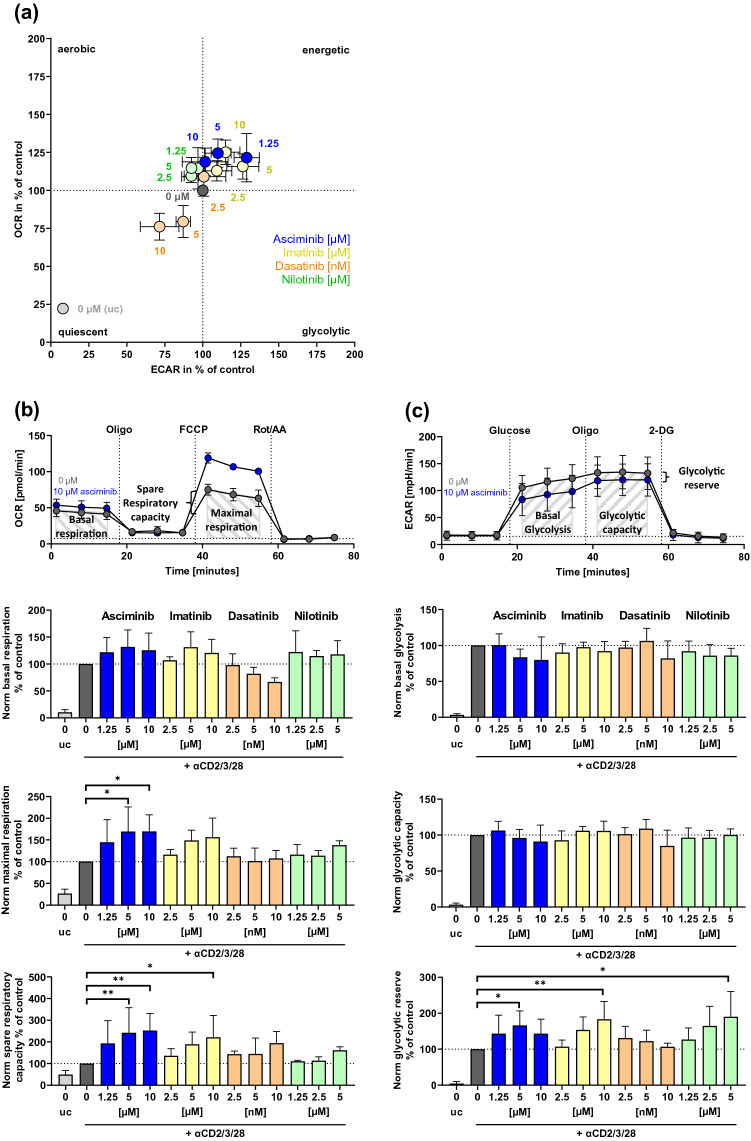
Metabolic flux analysis of TKI-treated T cells. Human T cells were treated with/without the TKIs asciminib (blue), imatinib (yellow), dasatinib (orange) or nilotinib (green), stimulated with/without αCD2/3/28-beads for 72 h and analyzed via metabolic flux analysis (*n* = 3–4 different donors, *n* = 7–8 different donors for controls). **a** Metabolic phenotype. Measuring of OCR and ECAR before addition of metabolic inhibitors. **b** Mitochondrial stress test. Oligomycin (Oligo), carbonyl cyanide-4 (trifluoromethoxy) phenylhydrazone (FCCP) and rotenone/antimycin A (Rot/AA) were successively added, OCR was measured and metabolic parameters (basal respiration, maximal respiration and spare respiratory capacity; exemplarily illustrated for αCD2/3/28-bead-stimulated control) were calculated. **c** Glycolysis stress test. Glucose, Oligo and 2-deoxy-d-glucose (2-DG) were successively added, ECAR was measured and metabolic parameters (basal glycolysis, glycolytic capacity, and glycolytic reserve; exemplarily illustrated for αCD2/3/28-bead-stimulated control) were calculated. Measurements were normalized to the respective TKI-untreated controls. *Norm* normalized, *uc* unstimulated control (no bead addition), *OCR* oxygen consumption rate, *ECAR* extracellular acidification rate, **p < 0.01, *p < 0.05

To further characterize the effects of asciminib on glucose metabolism, we focused on different metabolic parameters, associated with the processes of oxPhos and glycolysis by performing metabolic flux analyzes. We measured OCR (indicative for an aerobic metabolic phenotype) and ECAR (indicative for a glycolytic metabolic phenotype) before the addition of metabolic inhibitors (Fig. 4a). As expected, αCD2/3/28-stimulation increased the T cell metabolic activity from a quiescent state to a more energetic level, indicated by a strong rise of OCR- and ECAR levels. The metabolic phenotype of T cells treated with either asciminib, imatinib, or nilotinib had the trend toward being more energetic than the TKI-untreated control, while dasatinib-treatment nonsignificantly decreased OCR and ECAR down to 75%.

To investigate the effects of asciminib on oxPhos in the presence of metabolic inhibitors, we performed mitochondrial stress tests by successive injection of Oligo, FCCP and Rot/AA as previously described [21] and by simultaneously measuring OCR in order to calculate different metabolic parameters, which are shown in Fig. 4b. We found that asciminib increased the oxPhos potential of T cells, indicated by up to 69% increased maximal phosphorylation and 152% increased spare respiratory capacity at 10 µM. These effects were also comparable with those of imatinib. Additionally, dasatinib-treated T cells could compensate for the nonsignificant inhibitory effects on basal respiration after FCCP-injection.

Since parameters describing oxPhos and mitochondrial fitness (∆Ψm) were increased but glucose uptake of T cells was impaired by asciminib, we analyzed how glycolysis, an alternative way of ATP-production, is affected by TKIs. Therefore, we performed a glycolysis stress test by cultivating T cells in glucose- and pyruvate-free medium and injecting glucose first, followed by Oligo and 2-DG (Fig. 4c) as previously described [21, 23]. We simultaneously measured ECAR to calculate different glycolytic parameters. We found no significant changes in basal glycolysis although there was a marginal trend toward inhibition. The glycolytic capacity mostly remained stable, whereas glycolytic reserve was significantly increased by asciminib (166%, SD = 40 (5 µM)), imatinib (183%, SD = 50 (10 µM)), and nilotinib (190%, SD = 70 (5 µM)) compared to untreated control (100%).

## Discussion

T cells are critical to achieving TFR since they control residual CML cells resistant to TKI-therapy [1]. While ATP-pocket-binding TKIs have several inhibiting off-target effects on T cell function, we here investigated whether asciminib differentially affects T cell activity due to its unique characteristic as the first-in-class STAMP-inhibitor. Earlier publications showed that ATP-pocket binding TKIs reduced in vitro T cell proliferation, expression of the activation markers CD25 and CD69, and cytokine secretion [6–11]. In fact, this was in accordance with our data, whereas asciminib had less inhibitory effects on T cell activation. Co-stimulatory CD28 and the amino acid transporter and CD69-binding partner CD98 [24] were downregulated by all TKIs, suggesting an inhibition of intracellular T cell signaling and function.

PD-1 is an inhibitory receptor of the CD28 family and is known as a key player in the regulation of T cell activation- and exhaustion, effector T cell responses and T cell tolerance [25, 26]. Increased PD-1-levels on T cells in CML patients (mostly CD8+ T cells) have been described to correlate with potential adverse effects on the disease course [27, 28]. In our in vitro experiments, we found a stronger decrease of PD-1 induced by ATP-pocket-binding TKIs compared to asciminib, which might be advantageous because reduced PD-1-expression is thought to promote sustained DMR in CML [3]. Since PD-1 is upregulated during T cell stimulation, minor inhibition of PD-1 of stimulated T cells by asciminib might be directly connected to the reduced activation inhibition. Because TKI effects on T cell markers could be only observed on protein and not on RNA-level in our study, we assume that changes occur at the post-transcriptional level.

Several studies showed that the inhibition of T cell function by imatinib, dasatinib and nilotinib is also reflected by a decreased secretion of pro-inflammatory cytokines [7, 8, 10, 29] potentially affecting immune reconstitution and course of CML. Here, we observed similar inhibiting effects of asciminib on IFNγ, IL-6, and IL-17A. Moreover, as described in previous studies [7, 8, 29], we also discovered a strong IL-2 reduction elicited by ATP-pocket binding TKIs, which is in accordance with the reduced expression of the α-subunit of the IL-2 receptor (CD25) [30]. In contrast, we found IL-2-levels unchanged by asciminib, which also matches with the lower inhibition of CD25. Therefore, we conclude that there might be differences in the impact on IL-2-receptor-induced signaling between asciminib and the other TKIs. We suggest that normal IL-2 secretion by T cells in the presence of asciminib allows immune reconstitution since IL-2 is critical to the modulation of immune responses and differentiation of naïve CD4^+^ T cells into different subtypes (e.g., Th1 [31] or Th2 [32]), which requires further ex vivo patient data. Conversely to IL-2, decreased levels of IL-6 by asciminib and the other TKIs might be beneficial for the course of CML since IL-6-levels in the serum of CML patients were increased and it was additionally shown in a mouse model that IL-6 directly contributes to the development of CML [33, 34]. Reduced IL-6-levels were also previously described in CML patients under TKI-therapy [35]. The contribution of IFNγ to the course of CML was examined in a study by Busilacchi et al. at a murine CML model. It showed that the eradication of leukemic stem cells (LSC) depends on low IFNγ-levels, secreted by CD8^+^ CTL. However, CTL supported LSC proliferation and differentiation by the secretion of IFNγ [36]. Therefore, the role of TKI-induced changes in T cell IFNγ-levels during CML remains controversial. Concerning IL-17, a previous study demonstrated increased levels in the blood but not in the bone marrow of newly diagnosed patients with CML and imatinib-treated CML patients in CP [37]. This agrees with the absence of IL-17A-inhibition by imatinib in our study. There is lacking knowledge about the contribution of IL-17A to the course of CML and in vivo IL-17A levels in dasatinib-, nilotinib-, and asciminib-treated CML patients. However, our in vitro results suggest inhibiting effects of these TKI. Interestingly, in our analyses, imatinib did not inhibit IL-17A- and nilotinib did not affect IFNγ-secretion, which is not in line with previous findings [29] but this study used unstimulated T cells, while we focused on αCD2/3/28-stimulated T cells.

Central T cell processes including activation are associated with an increased energy demand for proliferation, growth, and exertion of effector functions. Activated T cells meet their energy needs, e.g., by increasing their aerobic glycolysis and degradation of glutamine. However, there is lacking knowledge about how the metabolism of T cells is affected by TKI-treatment. We observed impaired glucose uptake by all TKI-treated T cells, which conforms to our expectation, since co-stimulation of CD28, which was inhibited by all TKIs, is required for maximal glucose uptake [38]. Additionally, Glut-1 surface trafficking has been shown to be associated with CD28-expression [38]. This could explain the inhibition of Glut-1 in our experiments, which was significant for asciminib.

Beside glucose, intracellular and extracellular FAs are an important energy source for many functions of T cells. In our study, we found that FA uptake and CD36-expression were partially inhibited by asciminib and to a greater extent by dasatinib, indicating that the intracellular FA levels might be reduced.

Asciminib-induced inhibition of glucose and FA uptake had no inhibiting effect on oxPhos and glycolysis, which are both used for the production of ATP, suggesting that limited amounts of uptaken glucose/FA substrate were sufficient for T cells to maintain normal ATP-production. However, we observed an increased ΔΨm by asciminib and imatinib alike and a slight rise of mitochondrial superoxide levels by asciminib, which might be a sign of elevated electron transport and oxPhos in the mitochondria [22, 39]. Metabolic flux analyses showed that basal oxPhos and glycolysis were only marginally affected by asciminib, but T cells could strongly raise their maximal respiration, oxPhos and glycolysis potential, indicating that they could react flexibly on energy demand when treated with asciminib. In particular, an increased maximal respiration along with an increased spare respiratory capacity is often associated with elevated oxidation of substrate as a reaction to a higher demand for ATP or a higher substrate supply [40]. Similar assumptions apply to the glycolytic reserve since glycolysis is an essential and rapid process that provides these cells with energy precursors [41]. Memory and regulatory T cells (*T*_Regs_) mainly use oxPhos for meeting their energetic demands [42]. This would suggest an increased memory response to asciminib. Interestingly, in contrast to asciminib, dasatinib showed a weak inhibition of basal oxPhos the cells could compensate for by increasing their spare respiratory capacity. Generally, dasatinib seemed to have stronger immunosuppressive effects on T cell function and metabolism compared to asciminib and the other TKIs, which could be explained by a larger target spectrum but also by its increased binding affinity to its target kinases [5, 43].

This study shows new insights into functional changes in T cells induced by asciminib, which can be compared with previous in vitro data of ATP-pocket binding TKIs. By the increasing prescription of asciminib since its approval for medical use in the USA in October 2021 for adult patients, future research on patient material will gain relevance and will show which findings can be recapitulated in an ex vivo setting. Clinical trials have demonstrated efficacy of asciminib in patients with resistance to preceding TKI-therapies combined with moderate side effects [12, 44], but the effects on TFR are unknown and are currently examined in clinical studies (e.g., NCT05413915 and NCT04838041).

In summary, our results showed overlapping effects of the novel TKI asciminib and ATP-pocket binding TKIs on T cell function (e.g., proliferation inhibition) and also some differences like a reduced inhibition of early T cell activation and IL-2-secretion by asciminib, which might be beneficial for the T cells’ anti-CML properties. We also found inhibiting metabolic effects of TKIs, especially for dasatinib, but we demonstrated that the T cells had a flexible glucose metabolism and could even increase their oxPhos- and glycolytic potential. Due to its combination of promising on-target effects in first clinical studies and reduced off-target effects on T cells in our study, we expect a benefit of asciminib in bypassing TKI-resistances and simultaneously maintaining T cell-mediated control of residual CML cells to achieve TFR.

### Supplementary Information

Below is the link to the electronic supplementary material.

Supplementary file1 (DOCX 397 KB)

## Data Availability

Data will be provided upon request by the corresponding author.

## Abbreviations

**AA**: Antimycin A
**7AAD**: 7-Aminoactinomycin
**ATP**: Adenosine triphosphate
**BP**: Blast phase
**CD**: Cluster of differentiation
**CML**: Chronic myeloid leukemia
**CP**: Chronic phase
**CTL**: Cytotoxic T lymphocyte
**2-DG**: 2-Deoxy-d-glucose
**DMR**: Deep molecular response
**ECAR**: Extracellular acidification rate
**ELISA**: Enzyme-linked immunosorbent assay
**FA**: Fatty acid
**FACS**: Fluorescence-activated cell sorting
**FCCP**: Carbonyl cyanide-4 (trifluoromethoxy) phenylhydrazone
**FCS**: Fecal calf serum
**Glut**: Glucose transporter
**IFNγ**: Interferon gamma
**IL**: Interleukin
**LCFA**: Long-chain fatty acid
**MdFI**: Median fluorescence intensity
**OCR**: Oxygen consumption rate
**Oligo**: Oligomycin
**OxPhos**: Oxidative phosphorylation
**PBMCs**: Peripheral blood mononuclear cells
**PBS**: Phosphate-buffered saline
**PD-1**: Programmed cell death protein 1
**P/S**: Penicillin–Streptomycin
**ROS**: Reactive oxygen species
**Rot**: Rotenone
**STAMP**: Specifically targets the ABL myristoyl pocket
**TFR**: Treatment-free remission
**T**_**H**_** cell**: T-helper cell
**TKI**: Tyrosine kinase inhibitor
**TMRE**: Tetramethylrhodamine, ethyl ester
**T**_**regs**_: Regulatory T cells
**VPD450**: Violet Proliferation Dye 450
