# Supplementary material for: Effects of the STAMP-inhibitor asciminib on T cell activation and metabolic fitness compared to tyrosine kinase inhibition by imatinib, dasatinib, and nilotinib
Source: Cancer Immunol Immunother. 2023 Jan 5;72(6):1661–72. doi: 10.1007/s00262-022-03361-8 (PMC10198838; doi:10.1007/s00262-022-03361-8)
Supplement: Supplementary file 1 — Supplementary file1 (DOCX 397 KB) [file 262_2022_3361_MOESM1_ESM.docx]

**Supplementary information to:**

*Cancer Immunology, Immunotherapy* (submitted in 2022) – Lukas Häselbarth et al.

**Supplementary Table 1: Antibodies for flow cytometry.**

| Antibody | Fluorochrome | Clone | Isotype | Manufacturer |
| --- | --- | --- | --- | --- |
| CD3 | APC | UCHT1 | Mouse IgG1, κ | Biolegend |
| CD4 | FITC | RPA-T4 | Mouse IgG1, κ | Biolegend |
| CD4 | APC/Cy7 | OKT-4 | Mouse IgG2b, κ | Biolegend |
| CD8 | APC/Cy7 | HIT8a | Mouse IgG1, κ | Biolegend |
| CD8 | PE/Cy7 | SK1 | Mouse IgG1, κ | Biolegend |
| CD25 | PE/Cy7 | BC96 | Mouse IgG1, κ | Biolegend |
| CD28 | PerCP/Cy5.5 | CD28.2 | Mouse IgG1, κ | Biolegend |
| CD36 | PE | 5-271 | Mouse IgG2a,κ | Biolegend |
| CD69 | PerCP/Cy5.5 | FN50 | Mouse IgG1, k | Biolegend |
| CD98 | PE/Vio770 | REA387 | Recombinant Human IgG1 | Miltenyi Biotec |
| CD137 | PE/Cy7 | 4B4-1 | Mouse IgG1, k | Biolegend |
| PD-1 | PE | EH12.2H7 | Mouse IgG1, κ | Biolegend |
| Glut-1 | AF488 | 202915 | Mouse IgG2b | R&D Systems |

**Supplementary Figure 1: Therapeutical TKI-concentrations are subtoxic for T cells.** Human T cells were treated with the TKIs asciminib (blue), imatinib (yellow), dasatinib (orange) or nilotinib (green), stimulated with/without αCD2/3/28-beads for 72h and stained with antibodies against CD4/CD8 and 7-AAD/Annexin V. Cell viability was measured by flow cytometry (n=5 different donors).

**Supplementary Figure 2: TKI-induced changes in CD28, CD98, PD-1, CD25, CD69, and CD137 gene expression.** Human T cells were treated with 5 µM asciminib (blue), 5 µM imatinib (yellow), 5 nM dasatinib (orange) or 2.5 µM nilotinib (green) and stimulated with αCD2/3/28-beads for 72h. RNA was isolated and gene expression analyzed by RNA-sequencing. padj = adjusted p-value (n=3 different donors).

**Supplementary Figure 3: Imatinib, dasatinib, and nilotinib inhibit T cell proliferation.** Human T cells were treated with the TKIs imatinib (yellow), dasatinib (orange) or nilotinib (green) and stimulated with/without αCD2/3/28-beads for 72h. (a) Proliferation. VPD450-staining and measurement by FACs (n=5). (b) Cytokine secretion. Supernatants were analyzed for IFNγ, IL-2, IL-6 and IL-17A by ELISA. Measurements were normalized to the respective TKI-untreated controls (= 0% cytokine secretion, n=5-6 different donors). uc = unstimulated control (no bead addition), *** p < 0.001, ** p < 0.01, * p < 0.05.

**Supplementary Figure 3: Effects of TKIs on mitochondrial functions.** Human T cells were treated with the TKIs asciminib (blue), imatinib (yellow), dasatinib (orange) or nilotinib (green), stimulated with/without αCD2/3/28-beads for 72h and stained with TMRE (membrane potential), MitoTracker^TM^ Green FM (biomass) or MitoSOX^TM^ Red Mitochondrial Superoxide Indicator (superoxid generation). Expression (MdFI) of parameters were analyzed via flow cytometry (n=5-8 different donors). Measurements were normalized to the respective TKI-untreated controls. Norm = normalized, uc = unstimulated control (no bead addition), **p < 0.01, * p < 0.05.
